# Esophageal Inlet Patch and Linear IgA Disease in Upper Airway Stenosis: A Clinical Case Study

**DOI:** 10.1002/oto2.70198

**Published:** 2026-02-18

**Authors:** Justin Lau, Haley Howard, Jay Wasman, N. Scott Howard

**Affiliations:** ^1^ Case Western Reserve University School of Medicine Cleveland Ohio USA; ^2^ Department of Otolaryngology–Head and Neck Surgery University Hospitals Cleveland Medical Center Cleveland Ohio USA; ^3^ Department of Pathology University Hospitals Cleveland Medical Center Cleveland Ohio USA

**Keywords:** airway, inlet patch, laryngology, linear IgA disease, reflux, stenosis

An inlet patch (IP) is an abnormal area of heterotopic gastric mucosa in the esophagus typically located in the postcricoid region at or below the upper esophageal sphincter. While generally asymptomatic, symptoms may include dysphagia, esophagitis, stricture, and even upper airway disease.^1^ In this report, we present what we believe as the first case of IP associated with supraglottic stenosis and linear IgA disease. This case study uses de‐identified data that is exempt from Western Institutional Board review (IRB) as per Section §164.514(b) (1) of the HIPAA Privacy Rule.

## Case Report

A 74‐year‐old female presenting with dyspnea, throat tenderness, and dysphagia for solids was referred to our clinic from a local ENT. Prior history included esophageal stricture dilations, as well as coronary artery bypass graft and diabetes. She reported a family history of esophageal stenosis in her mother and several siblings.

**Figure 1:**
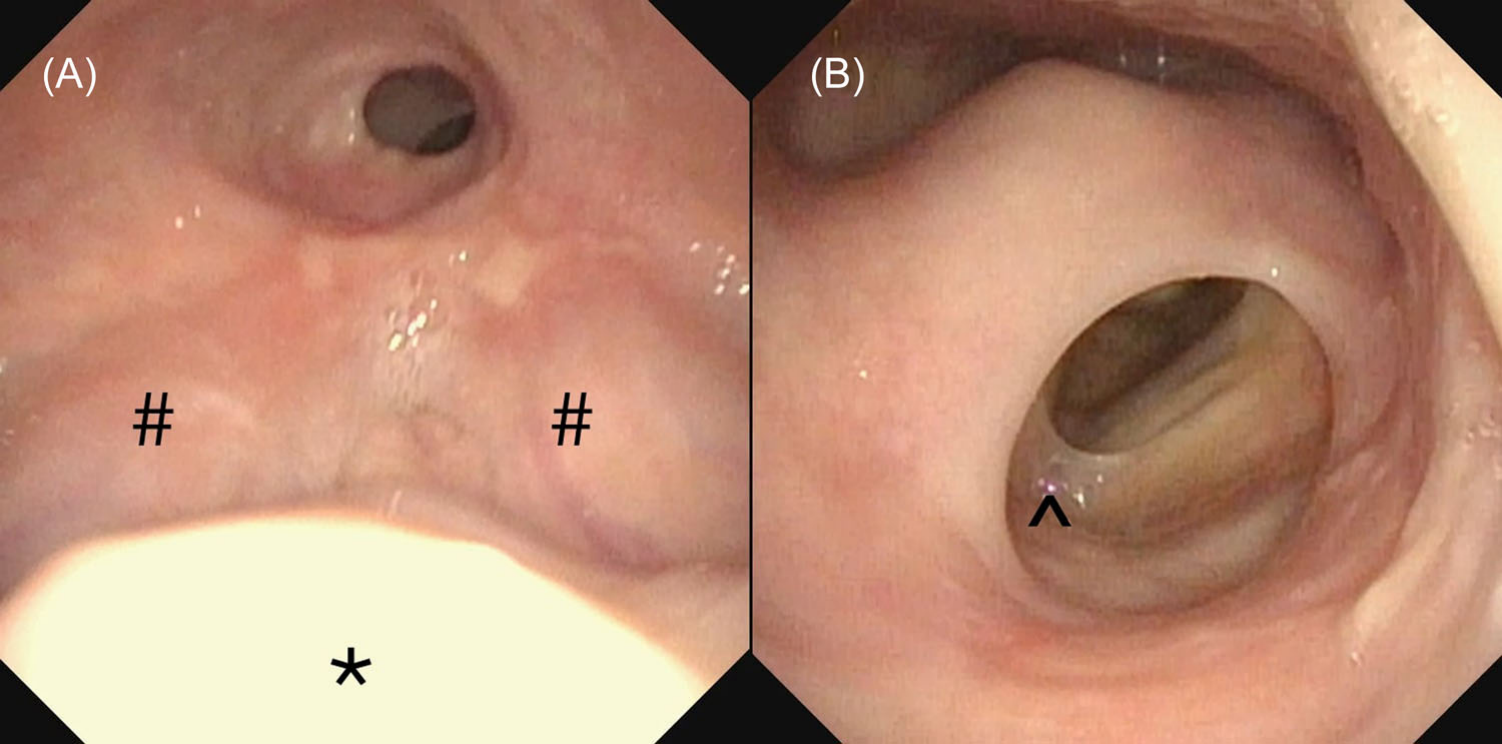
(A) Supraglottic stenosis. *: part of uvula. #: base of tongue. Note absence of epiglottis and 10 mm pharyngeal port. (B) Supraglottic and left hypopharyngeal stenosis just below (A) ^: anterior commissure web.

Flexible laryngoscopy demonstrated significant supraglottic and hypopharyngeal stenosis (Figure 1A and B). The epiglottis was completely imbedded within soft tissue scar. The opening from the oropharynx to the hypopharynx was measured at only 10 mm in diameter. Beyond the tongue base stenosis, there was complete absence of the left pyriform sinus and narrowed esophageal entry.

**Figure 2:**
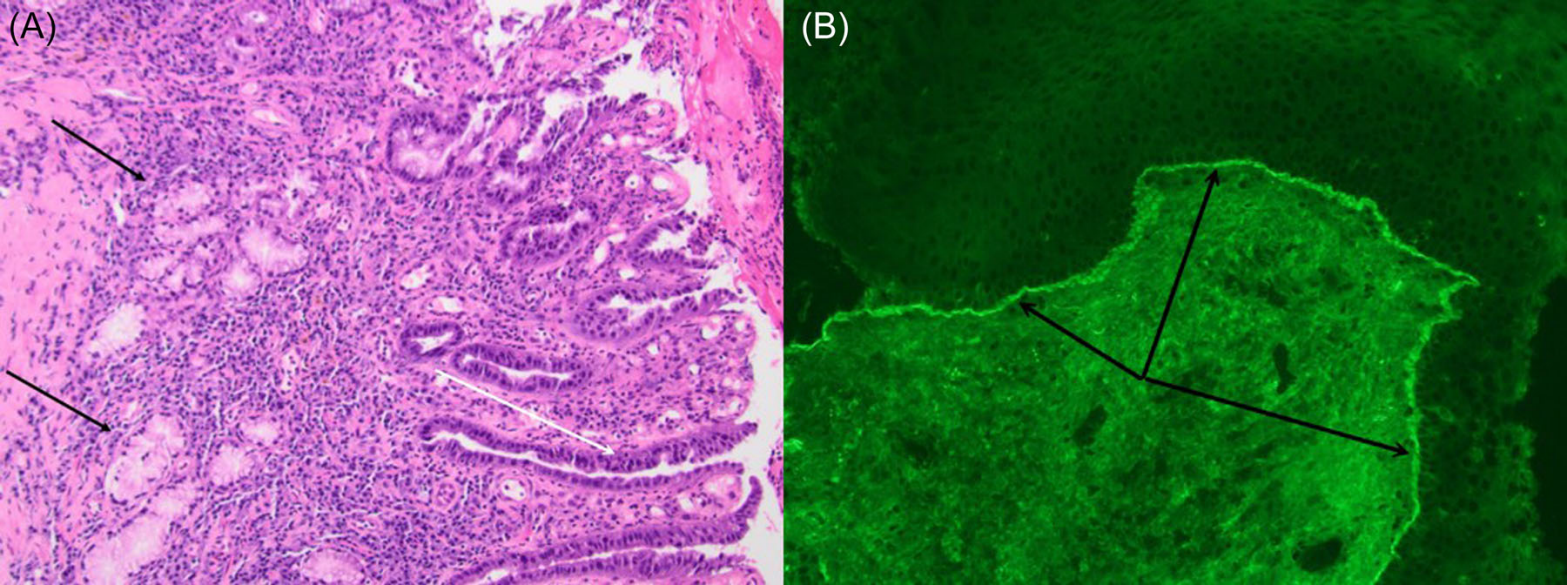
(A) Esophageal mass biopsy: surface glandular mucosa with mixed inflammatory reaction, reactive epithelial changes (white arrow), and deep mucous glands (black arrows) suggesting IP. (B) Hypopharyngeal biopsy: linear basement membrane deposition of IgA.

The patient underwent direct laryngoscopy with jet ventilation, balloon dilation, and CO_2_ laser ablation, opening the stenosis to improve breathing and swallowing. During the procedure, a raised salmon colored mass in the cervical esophagus was identified and removed. An IP was confirmed by surgical pathology (Figure 2A).

She was discharged after an overnight stay with significant improvement in diet tolerance and breathing. Postoperative modified barium swallow showed the epiglottis was partially revealed, but she had some penetration of thin liquids. At her one‐month follow‐up, she was tolerating a full diet with significant voice improvement. She was prescribed famotidine and Dexilant to address residual effects of the IP, which was not observed on in‐office esophagoscopy.

After three months, mild recurrence of scarring within the supraglottis and hypopharynx was observed; thus, she underwent repeat CO_2_ laser excision, balloon dilation, and steroid injection. Biopsies taken from the stenotic areas demonstrated chronic inflammatory infiltrate and granulation tissue. Linear IgA deposits along the basement membrane without IgG or C3 deposits were seen on pathology, suggesting linear IgA disease (LAD), a variant of mucous membrane pemphigoid (Figure 2B). She remained intervention‐free for 18 months with a touch up procedure performed to improve mild dysphagia from limited recurrence of hypopharyngeal scar.

## Discussion

Ectopic gastric mucosa can occur anywhere along and outside the gastrointestinal tract, but an IP specifically refers to heterotopic gastric mucosa at the upper esophagus.^1^ Most cases of IPs are asymptomatic, although aerodigestive symptoms can manifest. Some case reports have linked the IP to laryngeal pemphigus and laryngeal squamous cell carcinoma triggered by repeated acid exposure.^1^, ^2^ This is the first case of IP and LAD associated with upper airway and pharyngeal stenosis. LAD is a rare autoimmune mucocutaneous disease characterized by IgA autoantibodies targeting self‐antigens in the basement membrane.^3^ One report described fatal laryngeal scarring and stenosis from LAD, but evaluation for IP was not described.^4^ Although GERD has been associated with glottic scarring, we hypothesize that the presence of the IP, with subsequent elevated acid production acting as a caustic agent to promote severe scarring of the upper airway, may have limited contribution to the development of scarring or LAD.^5^ Our report demonstrates the possible severity of complications beyond dysphagia in patients with an IP and the importance of an otolaryngology evaluation, as prior endoscopies with GI missed the stenosis and IP in this patient due to poor visualization of the oropharynx and hypopharynx with flexible esophagoscopy. Additionally, this report adds to the spectrum of possible autoimmune diseases that may be associated with IPs. In patients with findings suggestive of upper digestive mucocutaneous autoimmune disease, we feel that esophagoscopy with narrow band imaging may assist with enhancing detection of an IP as removal may reduce incidence or recurrence of stenosis. This expanded perspective enhances clinical understanding and underscores the need for comprehensive evaluation in patients presenting with findings suggestive of pemphigoid, including inspection of the esophagus and hypopharynx for IPs and an in‐depth pathologic assessment that require close collaboration with the pathology team.

## Author Contributions

**Justin Lau,** conceptualization, investigation, draft preparation, review and editing; **Haley Howard,** conceptualization, investigation, draft preparation, review and editing; **Jay Wasman,** Investigation, resources, review and editing; **N. Scott Howard,** conceptualization, supervision, review and editing.

## Disclosures

### Competing interests

None.

### Funding source

None.
